# Certain Fractional Integral Formulas Involving the Product of Generalized Bessel Functions

**DOI:** 10.1155/2013/567132

**Published:** 2013-11-28

**Authors:** D. Baleanu, P. Agarwal, S. D. Purohit

**Affiliations:** ^1^Department of Chemical and Materials Engineering, Faculty of Engineering, King Abdulaziz University, P.O. Box 80204, Jeddah 21589, Saudi Arabia; ^2^Department of Mathematics and Computer Sciences, Faculty of Art and Sciences, Cankaya University, Ankara, Turkey; ^3^Institute of Space Sciences, P.O. Box MG-23, 76900 Magurele Bucharest, Romania; ^4^Department of Mathematics, Anand International College of Engineering, Jaipur 303012, India; ^5^Department of Basic-Sciences (Mathematics), College of Technology and Engineering, M.P. University of Agriculture and Technology, Udaipur 313001, India

## Abstract

We apply generalized operators of fractional integration involving Appell's function *F*_3_(·) due to Marichev-Saigo-Maeda, to the product of the generalized Bessel function of the first kind due to Baricz. The results are expressed in terms of the multivariable generalized Lauricella functions. Corresponding assertions in terms of Saigo, Erdélyi-Kober, Riemann-Liouville, and Weyl type of fractional integrals are also presented. Some interesting special cases of our two main results are presented. We also point out that the results presented here, being of general character, are easily reducible to yield many diverse new and known integral formulas involving simpler functions.

## 1. Introduction, Definitions, and Preliminaries

The fractional calculus is nowadays one of the most rapidly growing subject of mathematical analysis. It is a field of applied mathematics that deals with derivatives and integrals of arbitrary orders. The fractional integral operator involving various special functions has found significant importance and applications in various subfields of applicable mathematical analysis. Many applications of fractional calculus can be found in turbulence and fluid dynamics, stochastic dynamical system, plasma physics and controlled thermonuclear fusion, nonlinear control theory, image processing, nonlinear biological systems, astrophysics, and in quantum mechanics. Since last four decades, a number of workers like Love [1], McBride [2], Kalla [3, 4], Kalla and Saxena [5], Saigo [6, 7], Kilbas [8], Kilbas and Sebastian [9], Kiryakova [10, 11], Baleanu [12], Baleanu and Mustafa [13], Baleanu et al. [14], Baleanu et al. [15], Purohit and Kalla [16], Purohit [17], Agarwal [18–20] and Agarwal and Jain [21], and so forth have studied, in depth, the properties, applications, and different extensions of various operators of fractional calculus. A detailed account of fractional calculus operators along with their properties and applications can be found in the research monographs by Miller and Ross [22], Kiryakova [10], and so forth.

The computation of fractional derivatives (and fractional integrals) of special functions of one and more variables is important from the point of view of the usefulness of these results in the evaluation of generalized integrals and the solution of differential and integral equations. Motivated by these avenues of applications, a remarkably large number of fractional integral formulas involving a variety of special functions have been developed by many authors (see, e.g., [23–27]). Fractional integration formulae for the Bessel function and generalized Bessel functions are given recently by Kilbas and Sebastian [9], Saxena et al. [28], Malik et al. [29] and Purohit et al. [27]. A useful generalization of the Bessel function *w*_*ν*_(*z*) has been introduced and studied in [30–34]. Here we aim at presenting composition formula of Marichev-Saigo-Maeda fractional integral operators and the product of generalized Bessel function, which are expressed in terms of the multivariable generalized Lauricella functions. Some interesting special cases of our main results are also considered.

For our purpose, we begin by recalling some known functions and earlier works. This paper deals with two integral transforms defined for *x* > 0 and *α*, *α*′, *β*, *β*′, *γ* ∈ *ℂ* by

(1) (I0,+α,α′,β,β′,γf)(x)  =x−αΓ(γ)∫0x(x−t)γ−1t−α′F3(α,α′,β,β′;γ;  1−tx,1−xt)dx,(ℜ(γ)>0),

(2) (I0,−α,α′,β,β′,γf)(x)  =x−α′Γ(γ)∫x∞(t−x)γ−1t−αF3(α,α′,β,β′;γ;  1−xt,1−tx)dx,(ℜ(γ)>0).

These operators (integral transforms) were introduced by Marichev [35] as Mellin type convolution operators with a special function *F*_3_(·) in the kernel. These operators were rediscovered and studied by Saigo in [36] as generalization of the so-called Saigo fractional integral operators (see [37]). Such further properties as, for example, their relations with the Mellin transform and with the hypergeometric operators (or the Saigo fractional integral operators), together with their decompositional, operational and other properties in the McBride space *F*_*p*;*μ*_ (see [38]) were studied by Saigo and Maeda [39].

In (1) and (2) the symbol *F*_3_(·) denotes so-called 3rd Appell function (known also as Horn function) (see [40, page 413]):

(3) F3(α,α′;β,β′;η;x;y)  =∑m,n=0∞(α)m(α′)n(β)m(β′)n(η)m+nm!n!xmyn      ×(max⁡  {|x|,|y|}<1).

The properties of this function are discussed in [40, pages 412–415]. In particular, its relation to the Gauss hypergeometric function is presented as follows:

(4) F3(α,η−α;β,η−β;η;x;y)  =F12 (α,β;η;x+y−xy).

It is known that the 3rd Appell function cannot be expressed as a product of two _2_^^ *F*_1_ functions that satisfy pairs of linear partial differential equations of the second order.

We investigate compositions of integral transforms (1) and (2) with the product of generalized Bessel function of the first kind, *w*_*ν*_(*z*), which is defined for *z* ∈ *ℂ*∖{0} and *b*, *c*, *ν* ∈ *ℂ* with *ℜ*(*ν*)>−1 by the following series (see, e.g., [30, page 10, equation (1.15)]; for a very recent work, see also [31–33], [34, page 182, equation (2.2)], and [29, page 2, equation (8)]):

(5) $$\begin{array}{c} w_{\nu }\left(z\right)=\sum_{k=0}^{\infty }\frac{{\left(-1\right)}^{k}c^{k}{\left(z/2\right)}^{\nu +2k}}{k!\Gamma \left(\nu +k+\left(1+b\right)/2\right)}, \end{array}$$

where *ℂ* denotes the set of complex numbers and Γ(*z*) is the familiar Gamma function (see [41, Section 1.1]).

Then we show that the composition is expressed in terms of the multivariable generalized Lauricella functions due to Srivastava and Daoust (see, [42, page 454]) is a generalization of the Wright function _*p*_^ ^Ψ_*q*_ in several variables and defined by (see e.g., [43, page 107])

(6) Sq;q1;…;qnp;p1;…;pn[[(a)p:(αp′),…,(αpn)]:[(c′)p1:(γp1′)];…;[(c)p1n:(γp1n)];[(b)q:(βq′),…,(βqn)]:[(d′)q1:(δq1′)];…;[(d)q1n:(δq1n)];z1,…,zn]  =∑m1,…,mn=0∞A(m1,…,mn)∏j=1nzjmjmj!,

where

(7) A(m1,…,mn)=∏j=1pΓ(aj+∑j=1mmkαjk)∏j=1qΓ(bj+∑j=1mmkβjk) ×∏j=1n{∏j=1pkΓ(cjk+mkγjk)∏j=1qkΓ(djk+mkδjk)}.

The coefficients *α*_*j*_^*k*^ (*j* = 1,…, *p*), *β*_*j*_^*k*^ (*j* = 1,…, *q*), *γ*_*j*_^*k*^ (*j* = 1,…, *p*_*k*_), and *δ*_*j*_^*k*^ (*j* = 1,…, *q*_*k*_), for all *k* = 1,…, *n*, are real and positive and (*a*_*p*_) means the array of *p*-parameters *a*_1_,…, *a*_*p*_; with similar interpretations for (*b*_*p*_), (*γ*_*p*_1__^1^), (*α*_*p*_1__^1^), and so forth, and where (*a*)_*n*_ is the Pochhammer symbol defined (for *a* ∈ *ℂ*) by (see [41, page 2 and pages 4–6])

(8) (a)n:={1,(n=0),a(a+1)⋯(a+n−1),(n∈ℕ:={1,2,3,…})=Γ(a+n)Γ(a) (a∈ℂ∖ℤ0−),

and *ℤ*_0_^−^ denotes the set of nonpositive integers.

The paper is organized as follows. The composition formula of Marichev-Saigo-Maeda fractional integral operators (1) and (2) with the product of generalized Bessel function of the first kind (5) is proved in terms of the multivariable generalized Lauricella functions (6) in Sections 2 and 3, respectively. The corresponding results for the corresponding assertions in terms of Saigo, Erdélyi-Kober, Riemann-Liouville and Weyl type of fractional integrals are also presented in Sections 2 and 3. Special cases giving compositions of fractional integrals with the product of trigonometric functions and concluding remarks are considered in Section 4.

## 2. Left-Side Fractional Integration of Generalized Bessel Functions

Our results in this Section are based on the preliminary assertions giving composition formula of fractional integral (1) with a power function.

**Lemma 1 (Saigo and Maeda [39, Lemma 1])**

Let *α*, *α*′, *β*, *β*′, *γ* ∈ *ℂ* and if *ℜ*(*γ*) > 0, *ℜ*(*ρ*) > max⁡{0, *ℜ*(*α* + *α*′ + *β* − *γ*), *ℜ*(*α*′ − *β*′)}, then

(9) (I0,+α,α′,β,β′,γxρ−1)(x) =Γ(ρ)Γ(ρ+γ−α−α′−β)Γ(ρ+β′−α′)Γ(ρ+β′)Γ(ρ+γ−α−α′)Γ(ρ+γ−α′−β)  ×xρ+γ−α−α′−1.

The Marichev-Saigo-Maeda fractional integration (1) of product of generalized Bessel functions (5) and Binomial function is given by the following result.

**Theorem 2**

Let *α*, *α*′, *β*, *β*′, *σ*, *λ*, *γ*, *ν*_*j*_, *ρ*_*j*_, *b*, *c* ∈ *ℂ* and satisfying the inequalities

(10) ℜ(γ)>0,  ℜ(σ)>0,  ℜ(∑j=1nνj+b+12)>0,ℜ(σ+∑j=1nνjρj)  >max⁡[0,ℜ(α+α′+β−γ),ℜ(α′−β′)],

then for *x* > 0, |*ax*/*b* | <1, one has

(11) (I0,+(α,α′,β,β′,γ)[tσ−1(b−at)−λ  ∏j=1nwνj(ajtρj)])(x)=xσ+γ−α−α′−1Γ(λ)bλ∏j=1n(ajxρj2)νjS3;1;…;1;03;0;…;0;1[A,C,E:G;B,D,F:H;−an2x2ρn4,…,−an2x2ρn4,axb],

where *A*, *B*, *C*, *D*, *E*, *F*, *G*, and *H* are given by the following

(12) $$\begin{array}{c} A=\left[\left(\sigma +\sum_{j=1}^{n}\nu_{j}\rho_{j}\right):\left(2\rho_{1}\right),\ldots ,\left(2\rho_{n}\right),\left(1\right)\right], \end{array}$$

(13) $$\begin{array}{c} B=\left[\left(\sigma +\sum_{j=1}^{n}\nu_{j}\rho_{j}+\beta^{\prime }\right):\left(2\rho_{1}\right),\ldots ,\left(2\rho_{n}\right),\left(1\right)\right], \end{array}$$

(14) C=[(σ+∑j=1nνjρj+γ−β−α−α′):(2ρ1),…,(2ρn),(1)],

(15) D=[(σ+∑j=1nνjρj+γ−α−α′):(2ρ1),…,(2ρn),(1)],

(16) $$\begin{array}{c} E=\left[\left(\sigma +\sum_{j=1}^{n}\nu_{j}\rho_{j}+\beta^{\prime }-\alpha^{\prime }\right):\left(2\rho_{1}\right),\ldots ,\left(2\rho_{n}\right),\left(1\right)\right], \end{array}$$

(17) F=[(σ+∑j=1nνjρj+γ−α′−β):(2ρ1),…,(2ρn),(1)],

(18) $$\begin{array}{c} G=-;\ldots ;-;\left[\lambda :1\right], \end{array}$$

(19) $$\begin{array}{c} H=\left[\nu_{1}+\frac{1+b}{2}:k_{1}\right];\ldots ;\left[\nu_{n}+\frac{1+b}{2}:k_{n}\right];\;0. \end{array}$$

**Proof**

For sake of convenience, let the left-hand side of the (11) be denoted by *ℐ*. Using definition (5) and the binomial expansion, namely,

(20) $$\begin{array}{c} {\left(b-at\right)}^{-\lambda }=b^{-\lambda }\sum_{k=0}^{\infty }\frac{{\left(\lambda \right)}_{k}}{k!}{\left(\frac{at}{b}\right)}^{k}\;\left|\frac{at}{b}\right|<1, \end{array}$$

we find

(21) ℐ=(I0,+(α,α′,β,β′,γ)[tσ−1(b−at)−λ  ∏j=1nwνj(ajtρj)])(x)=(I0,+(α,α′,β,β′,γ)   ×[tσ−1(b−λ∑k=0∞(λ)kk!(atb)k)    ×(∑k1=0∞(−1)k1(c)k1(a1tρ1/2)ν1+2k1k1!Γ(ν1+k1+(b+1)/2))    ×⋯×(∑kn=0∞(−1)kn(c)kn(antρn/2)νn+2knkn!Γ(νn+kn+((b+1)/2)))])(x).

Following the convergence condition of Theorem 2, for any *k* ∈ *ℕ*_0_,

(22) ℜ(σ+k+∑j=1nνjρj+2∑j=1nνjkj)  ≥ℜ(σ+∑j=1nνjρj)  >max⁡⁡[0,ℜ(α+α′+β−γ),ℜ(α′−β′)]

and then changing the order of integration and summation, we obtain

(23) ℐ=b−λ∑k,k1,…,kn=0∞(λ)k(−1)k1(c)k1(a1/2)ν1+2k1k!k1!Γ(ν1+k1+(b+1)/2)(ab)k      ×⋯(−1)kn(c)kn(an/2)νn+2knkn!Γ(νn+kn+(b+1)/2)      ×(I0,+(α,α′,β,β′,γ)       ×{tσ+k+ν1ρ1+⋯+νnρn+2ν1k1+⋯+2νnkn−1})(x).

Now on applying Lemma 1 and using (9) with *ρ* replaced by (*σ* + *k* + *ν*_1_ *ρ*_1_ + ⋯+*ν*_*n*_ *ρ*_*n*_ + 2*ν*_1_ *k*_1_ + ⋯+2*ν*_*n*_ *k*_*n*_), we obtain

(24) ℐ=xσ+γ−α−α′−1Γ(λ)bλ ×∏j=1n(ajxρj2)νj   ×∑k,k1,…,kn=0∞(Γ(λ+k)Γ(σ+k+∑j=1nνjρj+2∑j=1nνjkj))  ×(Γ(σ+β′+k+∑j=1nνjρj+2∑j=1nνjkj))−1  ×(Γ(σ+γ−α−α′−β+k+∑j=1nνjρj+2∑j=1nνjkj)  ×Γ(σ+β′−α′+k+∑j=1nνjρj+2∑j=1nνjkj))  ×(Γ(σ+γ−α−α′+k+∑j=1nνjρj+2∑j=1nνjkj)  ×Γ(σ+γ−α′−β+k+∑j=1nνjρj+2∑j=1nνjkj))−1  ×1Γ(ν1+k1+(b+1)/2)⋯Γ(νn+kn+(b+1)/2)  ×−(a1x2ρ1/4)k1k1!×⋯×−(anx2ρn/4)knkn!×(ax/b)kk!.

This, in accordance with (6), gives the required result (11). This completed the proof of the Theorem 2.

On setting *α*′ = 0 in Theorem 2, we get the following Saigo hypergeometric fractional image of the product of generalized Bessel function of the first formula kind *w*_*ν*_(*z*).

**Corollary 3**

If *α*, *β*, *β*′, *σ*, *λ*, *γ*, *ν*_*j*_, *ρ*_*j*_, *b*, *c* ∈ *ℂ* and *x* > 0, |*ax*/*b*| < 1

(25) $$\begin{array}{c} ℜ\left(\gamma \right)>0,\;\;ℜ\left(\sigma \right)>0,\;\;ℜ\left(\sum_{j=1}^{n}\nu_{j}+\frac{b+1}{2}\right)>0,\\ ℜ\left(\sigma +\sum_{j=1}^{n}\nu_{j}\rho_{j}\right)>0, \end{array}$$

then there hold the results:

(26) (I0,+(γ,α−γ,−β)[tσ−1(b−at)−λ  ∏j=1nwνj(ajtρj)])(x)=xσ+γ−α−1Γ(λ)bλ∏j=1n(ajxρj2)νjS2;1;…;1;02;0;…;0;1[A,C:G;D,F:H;−a12x2ρ14,…,−an2x2ρn4,axb],

where *A*, *C*, *D*, *F*, *G*, and *H* are given by (12), (14), (15), (17), (18), and (19), respectively.

Again, on letting *α*′ = 0 and *α* = 0 in Theorem 2, we get the Riemann-Liouville fractional image of the product of generalized Bessel function of the first formula kind; asserted by the following corollary.

**Corollary 4**

Let *β*, *β*′, *σ*, *λ*, *γ*, *ν*_*j*_, *ρ*_*j*_, *b*, *c* ∈ *ℂ* and *x* > 0, |*ax*/*b*| < 1, and

(27) $$\begin{array}{c} ℜ\left(\gamma \right)>0,\;\;ℜ\left(\sigma \right)>0,\;\;ℜ\left(\sum_{j=1}^{n}\nu_{j}+\frac{b+1}{2}\right)>0,\\ ℜ\left(\sigma +\sum_{j=1}^{n}\nu_{j}\rho_{j}\right)>0. \end{array}$$

Then, one has

(28) (I0,+(γ)[tσ−1(b−at)−λ  ∏j=1nwνj(ajtρj)])(x)=xσ+γ−1Γ(λ)bλ∏j=1n(ajxρj2)νjS1;1;…;1;01;0;…;0;1[A:G;D:H;−a12x2ρ14,…,−an2x2ρn4,axb],

where *A*, *D*, *G*, and *H* are given by (12), (15), (18), and (19), respectively.

## 3. Right-Side Fractional Integration of Generalized Bessel Functions

In the sequel, we use the following results.

**Lemma 5 (Saigo and Maeda [39, Lemma 2])**

Let *α*, *α*′, *β*, *β*′, *γ* ∈ *ℂ* and if *ℜ*(*γ*) > 0, *ℜ*(*ρ*) < 1 + min⁡{*ℜ*(−*β*), *ℜ*(*α* + *α*′ − *γ*), *ℜ*(*α* + *β*′ − *γ*)}, then

(29) (I0,−α,α′,β,β′,γxρ−1)(x)  =(Γ(1−ρ−β)Γ(1−ρ−γ+α+α′)     ×Γ(1−ρ+α+β′−γ))   ×(Γ(1−ρ)Γ(1−ρ+α+α′+β′−γ)     ×Γ(1−ρ+α−β))−1xρ+γ−α−α′−1.

The Marichev-Saigo-Maeda fractional integration (2) of product of generalized Bessel functions (5) and Binomial function is given by the following result.

**Theorem 6**

Let *α*, *α*′, *β*, *β*′, *σ*, *λ*, *γ*, *ν*_*j*_, *ρ*_*j*_, *b*, *c* ∈ *ℂ* and *x* > 0, |*a*/*bx* | <1 such that

(30) ℜ(γ)>0,  ℜ(σ)>0,  ℜ(∑j=1nνj+b+12)>0,ℜ(σ+∑j=1nνjρj)  <1+min⁡{ℜ(−β),ℜ(α+α′−γ),ℜ(α+β′−γ)}.

Then, one has

(31) (I0,−(α,α′,β,β′,γ)[tσ−1(b−at)−λ  ∏j=1nwνj(ajtρj)])(x)=xσ+γ−α−α′−1Γ(λ)bλ∏j=1n(aj2xρj)νjS3;1;…;1;03;0;…;0;1[A′,C′,E′:G;B′,D′,F′:H;−a124x2ρ1,…,−an24x2ρn,abx],

where *A*′, *B*′, *C*′, *D*′, *E*′, and *F*′ are given by the following:

(32) $$\begin{array}{c} A^{\prime }=\left[\left(1-\sigma -\beta +\sum_{j=1}^{n}\nu_{j}\rho_{j}\right):\left(2\rho_{1}\right),\ldots ,\left(2\rho_{n}\right),\left(1\right)\right], \end{array}$$

(33) $$\begin{array}{c} B^{\prime }=\left[\left(1-\sigma +\sum_{j=1}^{n}\nu_{j}\rho_{j}\right):\left(2\rho_{1}\right),\ldots ,\left(2\rho_{n}\right),\left(1\right)\right], \end{array}$$

(34) C′=[(1−σ−γ+α+α′+∑j=1nνjρj):(2ρ1),…,  (2ρn),(1)],

(35) $$\begin{array}{c} D^{\prime }=\left[\left(1-\sigma +\alpha -\beta +\sum_{j=1}^{n}\nu_{j}\rho_{j}\right):\left(2\rho_{1}\right),\ldots ,\left(2\rho_{n}\right),\left(1\right)\right], \end{array}$$

(36) E′=[(1−σ−γ+α+β′+∑j=1nνjρj):(2ρ1),…,  (2ρn),(1)],

(37) F′=[(1−σ−γ+α+α′+β′+∑j=1nνjρj):(2ρ1),…,  (2ρn),(1)],

and *G*, *H* are given by (18) and (19), respectively.

**Proof**

For convenience, let the left-hand side of the (31) be denoted by *𝒥*. Applying definition (5) and the binomial expansion for (*b* − *at*)^−*λ*^ and then changing the order of integration and summation, we find

(38) 𝒥=b−λ∑k,k1,…,kn=0∞(λ)k(−1)k1(c)k1(a1/2)ν1+2k1k!k1!Γ(ν1+k1+(b+1)/2)×(ab)k⋯(−1)kn(c)kn(an/2)νn+2knkn!Γ(νn+kn+(b+1)/2)×(I0,+(α,α′,β,β′,γ)×{tσ−k−ν1ρ1−⋯−νnρn−2ν1k1−⋯−2νnkn−1})(x).

Following the convergence condition of Theorem 6, for any *k* ∈ *ℕ*_0_

(39) ℜ(σ−k−∑j=1nνjρj−2∑j=1nνjkj)  ≤ℜ(σ−∑j=1nνjρj)  <1+min⁡⁡{ℜ(−β),ℜ(α+α′−γ),ℜ(α+β′−γ)}.

Now, on making use of Lemma 5 and (9), we obtain

(40) 𝒥=xσ+γ−α−α′−1Γ(λ)bλ ×∏j=1n(aj2xρj)νj    ×∑k,k1,…,kn=0∞(Γ(λ+k)Γ(1−σ−β+k+∑j=1nνjρj+2∑j=1nνjkj))   ×(Γ(1−σ−γ+α+α′+β′+k   +∑j=1nνjρj+2∑j=1nνjkj))−1   ×(Γ(1−σ−γ+α+α′+k   +∑j=1nνjρj+2∑j=1nνjkj)   ×Γ(1−σ−γ+α+β′+k   +∑j=1nνjρj+2∑j=1nνjkj))   ×(Γ(1−σ+k+∑j=1nνjρj+2∑j=1nνjkj)   ×Γ(1−σ+α−β+k+∑j=1nνjρj+2∑j=1nνjkj))−1   ×1Γ(ν1+k1+(b+1)/2)⋯Γ(νn+kn+(b+1)/2) −(a1/4x2ρ1)k1k1!⋯−(an/4x2ρn)knkn!(a/bx)kk!.

This, in accordance with (6), gives the required result (31). This completed the proof of Theorem 6.

On taking *α*′ = 0 in Theorem 6, we get the right-sided Saigo hypergeometric fractional image of the product of generalized Bessel function of the first formula kind, *w*_*ν*_(*z*) as follows.

**Corollary 7**

Let *α*, *β*, *β*′, *σ*, *λ*, *γ*, *ν*_*j*_, *ρ*_*j*_, *b*, *c* ∈ *ℂ* and *x* > 0, |*a*/*bx*| < 1, and

(41) $$\begin{array}{c} ℜ\left(\gamma \right)>0,\;\;ℜ\left(\sigma \right)>0,\;\;ℜ\left(\sum_{j=1}^{n}\nu_{j}+\frac{b+1}{2}\right)>0,\\ ℜ\left(\sigma +\sum_{j=1}^{n}\nu_{j}\rho_{j}\right)>0. \end{array}$$

Then

(42) (Ix,∞(γ,α−γ,−β)[tσ−1(b−at)−λ  ∏j=1nwνj(ajtρj)])(x)=xσ+γ−α−α′−1Γ(λ)bλ∏j=1n(aj2xρj)νjS2;1;…;1;02;0;…;0;1[A′,C′:G;B′,D′:H;−a124x2ρ1,…,−an24x2ρn,abx],

where *A*′, *B*′, *C*′, *D*′, *G* and *H* are given by (32), (33), (34), (35), (18), and (19), respectively.

Again, on setting *α*′ = 0 and *α* = 0 in Theorem 6, we get the following image formula for the product of generalized Bessel function of the first kind involving Riemann-Liouville type fractional integral operator.

**Corollary 8**

Let *β*, *β*′, *σ*, *λ*, *γ*, *ν*_*j*_, *ρ*_*j*_, *b*, *c* ∈ *ℂ* and *x* > 0, |*a*/*bx*| < 1, and

(43) $$\begin{array}{c} ℜ\left(\gamma \right)>0,\;\;ℜ\left(\sigma \right)>0,\;\;ℜ\left(\sum_{j=1}^{n}\nu_{j}+\frac{b+1}{2}\right)>0,\\ ℜ\left(\sigma +\sum_{j=1}^{n}\nu_{j}\rho_{j}\right)>0. \end{array}$$

Then

(44) (Ix,∞(γ)[tσ−1(b−at)−λ  ∏j=1nwνj(ajtρj)])(x)=xσ+γ−α−α′−1Γ(λ)bλ∏j=1n(aj2xρj)νjS1;1;…;1;01;0;…;0;1[C′:G;B′:H;−a124x2ρ1,…,−an24x2ρn,abx],

where *B*′, *C*′, *G*, and *H* are given by (33), (34), (18), and (19), respectively.

## 4. Consequence Results and Concluding Remarks

In this section, we briefly consider another variation of the results derived in the preceding sections. Bessel functions are important special functions that appear widely in science and engineering. Bessel functions of the first kind *J*_*ν*_(*z*) are oscillatory and may be regarded as generalizations of trigonometric functions. Indeed, for large argument (*z* ≥ 1) the function $\sqrt{\pi z/2}J_{\nu }(z)$ is well approximated by the trigonometric function cos⁡(*z* − *πν*/2 − *π*/4). Similarly, modified Bessel functions of the first kind *I*_*ν*_(*z*), which are Bessel functions of imaginary argument, may be regarded as generalization of exponentials. Exponential functions have the unique and special property that they are particularly easy to multiply and to raise to powers: *e* ^*az*^ *e* ^*bz*^ = *e* ^(*a*+*b*)*z*^ and (*e* ^*z*^)^*r*^ = *e* ^*rz*^. Further, it can be easily seen that for *c* = 1 and *b* = 1, the Generalized Bessel function of the first kind (5) reduces to *J*_*ν*_(*z*), and when *c* = −1 and *b* = 1 the function *w*_*ν*_(*z*) becomes *I*_*ν*_(*z*). Similarly, when *c* = 1 and *b* = 2, the function *w*_*ν*_(*z*) reduces to $2j_{\nu }/\sqrt{\pi }$, while if *c* = −1 and *b* = 2, then *w*_*ν*_(*z*) becomes $2i_{\nu }/\sqrt{\pi }$. In the sequel from (5), we have *w*_*ν*_(0) = 0. Therefore, the results presented in this paper are easily converted in terms of the various special Bessel functions after some suitable parametric replacement.

For example, if we set *ν* = −*b*/2, then the generalized Bessel function *w*_*ν*_(*z*) in (5) have following relation with cosine function when *c* is replaced by *c* ^2^ (see, e.g., [29]):

(45) $$\begin{array}{c} w_{-b/2,c^{2}}\left(z\right)={\left(\frac{2}{z}\right)}^{b/2}\frac{\cos cz}{\sqrt{\pi }}. \end{array}$$

While if *ν* = −*b*/2 and *c* = −*c* ^2^, *w*_*ν*_(*z*) in (5) have relation with hyperbolic cosine function as (see, e.g., [29])

(46) $$\begin{array}{c} w_{-b/2,-c^{2}}\left(z\right)={\left(\frac{2}{z}\right)}^{b/2}\frac{\cosh cz}{\sqrt{\pi }}. \end{array}$$

In the sequel, if we set *ν* = 1 − *b*/2, then the generalized Bessel function *w*_*ν*_(*z*) in (5) have following relation with sine function when *c* is replaced by *c* ^2^ (see, e.g., [29]):

(47) $$\begin{array}{c} w_{1-b/2,c^{2}}\left(z\right)={\left(\frac{2}{z}\right)}^{b/2}\frac{\sin cz}{\sqrt{\pi }}. \end{array}$$

Further, if we put *ν* = 1 − *b*/2 and *c* = −*c* ^2^ in (5), we have the following relation with *w*_*ν*_(*z*) and hyperbolic sine function as (see, e.g., [29])

(48) $$\begin{array}{c} w_{1-b/2,-c^{2}}\left(z\right)={\left(\frac{2}{z}\right)}^{b/2}\frac{\sinh cz}{\sqrt{\pi }}. \end{array}$$

Now, by virtue of the relations (45) to (48), one can easily derive the Marichev-Saigo-Maeda type fractional integrals involving product of trigonometric functions. Therefore, we omit the details of these results.

Further, it is interesting to observe that, if we set at *α*′ = 0, the results given by Malik et al. [29, pages 5 and 8, Theorems 4.1 and 5.1] follow as special cases of our main results. Again, if we set *λ* = 0 and *b* = *c* = 1, then our main results (11) and (31) follow the kown results due to [28]. Finally, if we set *λ* = 0, *n* = *b* = *c* = 1 and give some suitable parametric replacement in the main results (11) and (31), we can arrive at the known results due to Purohit et al. [27] in a slightly different notation.

Fractional integral formulas involving products of Bessel functions have been developed and play an important role in several physical problems. In fact, Bessel functions are playing the important role in studying solutions of differential equations, integral equations [44], and they are associated with a wide range of problems in important areas of mathematical physics, like problems of acoustics, radio-physics, hydrodynamics, and atomic and nuclear physics. These considerations have led various workers in the field of special functions for exploring the possible extensions and applications for the Bessel functions. Among many properties of Bessel functions, they also have investigated some possible extensions of the Bessel functions. The generalized Bessel function defined by (5), possess the advantage that a number of Bessel functions, trigonometric functions and hyperbolic functions happen to be the particular cases of this function. Therefore, we conclude this paper with the remark that, the results deduced above are significant and can lead to yield numerous other fractional integrals involving various Bessel functions and trigonometric functions by the suitable specializations of arbitrary parameters in the theorems. More importantly, they are expected to find some applications to the solutions of fractional differential and integral equations. The results thus derived in this paper are general in character and likely to find certain applications in the theory of special functions.

## References

[1] Love ER (1967). Some integral equations involving hypergeometric functions. *Proceedings of the Edinburgh Mathematical Society*.

[2] McBride AC (1982). Fractional powers of a class of ordinary differential operators. *Proceedings of the London Mathematical Society*.

[3] Kalla SL (1969). Integral operators involving Fox’s *H*-function. *Acta Mexicana de Ciencia y Tecnología*.

[4] Kalla SL (1969). Integral operators involving Fox’s *H*-function. II.

[5] Kalla SL, Saxena RK (1969). Integral operators involving hypergeometric functions. *Mathematische Zeitschrift*.

[6] Saigo M (1978). A remark on integral operators involving the Gauss hypergeometric functions. *Mathematical Reports of College of General Education. Kyushu University*.

[7] Saigo M (1979). A certain boundary value problem for the Euler-Darboux equation. *Mathematica Japonica*.

[8] Kilbas AA (2005). Fractional calculus of the generalized Wright function. *Fractional Calculus & Applied Analysis*.

[9] Kilbas AA, Sebastian N (2008). Generalized fractional integration of Bessel function of the first kind. *Integral Transforms and Special Functions*.

[10] Kiryakova V (1994). *Generalized Fractional Calculus and Applications*.

[11] Kiryakova V (2008). A brief story about the operators of the generalized fractional calculus. *Fractional Calculus & Applied Analysis*.

[12] Baleanu D (2009). About fractional quantization and fractional variational principles. *Communication in Nonlinear Sciences and Numerical Simulation*.

[13] Baleanu D, Mustafa OG (2010). On the global existence of solutions to a class of fractional differential equations. *Computers & Mathematics with Applications*.

[14] Baleanu D, Mustafa OG, Agarwal RP (2010). On the solution set for a class of sequential fractional differential equations. *Journal of Physics A*.

[15] Baleanu D, Diethelm K, Scalas E, Trujillo JJ (2012). *Fractional Calculus Models and Numerical Methods*.

[16] Purohit SD, Kalla SL (2011). On fractional partial differential equations related to quantum mechanics. *Journal of Physics A*.

[17] Purohit SD (2013). Solutions of fractional partial differential equations of quantum mechanics. *Advances in Applied Mathematics and Mechanics*.

[18] Agarwal P (2013). Fractional integration of the product of two multivariables *H*-function and a general class of polynomials. *Advances in Applied Mathematics and Approximation Theory*.

[19] Agarwal P (2012). Further results on fractional calculus of Saigo operators. *Applications and Applied Mathematics*.

[20] Agarwal P (2012). Generalized fractional integration of the $\bar{H}$ function. *Le Matematiche*.

[21] Agarwal P, Jain S (2011). Further results on fractional calculus of Srivastava polynomials. *Bulletin of Mathematical Analysis and Applications*.

[22] Miller KS, Ross B (1993). *An Introduction to the Fractional Calculus and Fractional Differential Equations*.

[23] Agarwal P, Purohit SD (2013). The unified pathway fractional integral formulae. *Journal of Fractional Calculus and Applications*.

[24] Baleanu D, Mustafa OG, O’Regan D (2013). A uniqueness criterion for fractional differential equations with Caputo derivative. *Nonlinear Dynamics*.

[25] Purohit SD, Suthar DL, Kalla SL (2010). Some results on fractional calculus operators associated with the *M*-function. *Hadronic Journal*.

[26] Purohit SD, Kalla SL, Suthar DL (2011). Fractional integral operators and the multiindex Mittag-Leffler functions. *Scientia A*.

[27] Purohit SD, Suthar DL, Kalla SL (2012). Marichev-Saigo-Maeda fractional integration operators of the Bessel function. *Le Matematiche*.

[28] Saxena RK, Ram J, Kumar D Generalized fractional integration of the product of Bessel functions of the first kind.

[29] Malik P, Mondal SR, Swaminathan A (2011). *Fractional Integration of Generalized Bessel Function of the First Kind*.

[30] Baricz Á (2010). *Generalized Bessel Functions of the First Kind*.

[31] Baricz Á (2006). Geometric properties of generalized Bessel functions of complex order. *Mathematica*.

[32] Baricz Á (2008). Geometric properties of generalized Bessel functions. *Publicationes Mathematicae Debrecen*.

[33] Baricz Á (2008). Jordan-type inequalities for generalized Bessel functions. *Journal of Inequalities in Pure and Applied Mathematics*.

[34] Mondal SR, Swaminathan A (2012). Geometric properties of generalized Bessel functions. *Bulletin of the Malaysian Mathematical Sciences Society*.

[35] Marichev OI (1974). Volterra equation of Mellin convolution type with a Horn function in the kernel. *Izvestiya Akademii Nauk SSSR*.

[36] Saigo M On generalized fractional calculus operators.

[37] Kiryakova V (2006). On two Saigo’s fractional integral operators in the class of univalent functions. *Fractional Calculus & Applied Analysis*.

[38] McBride AC (1979). *Fractional Calculus and Integral Transforms of Generalized Functions*.

[39] Saigo M, Maeda N, Rusev P, Dimovski I, Kiryakova V (1998). More generalization of fractional calculus. *Transform Methods & Special Functions*.

[40] Olver FWJ, Lozier DW, Boisvert RF, Clark CW (2010). *NIST Handbook of Mathematical Functions*.

[41] Srivastava HM, Choi J (2012). *Zeta and q-Zeta Functions and Associated Series and Integrals*.

[42] Srivastava HM, Daoust MC (1969). Certain generalized Neumann expansions associated with the Kampé de Fériet function. * Koninklijke Nederlandse Akademie van Wetenschappen*.

[43] Exton H (1976). *Multiple Hypergeometric Functions and Applications*.

[44] Choi J, Agarwal P (2013). Certain unified integrals associated with Bessel functions. *Boundary Value Problems*.

